# Supplementary material for: Network Meta‐Analysis of Survival Outcomes With Non‐Proportional Hazards Using Flexible M‐Splines
Source: Stat Med. 2026 Aug 10;45(18-19):e70695. doi: 10.1002/sim.70695 (PMC13457511; doi:10.1002/sim.70695)
Supplement: Supplementary file 1 [file SIM-45-0-s001.pdf]

C ADDITIONAL TABLES AND FIGURES FOR NSCLC ANALYSIS

TABLE C.1 Baseline characteristics for the four studies included in the analysis of progression free survival with non-small cell lung cancer.

	ERACLE		IMpower150		KEYNOTE189		PRONOUNCE	
Covariate	BCP (N = 58)	PCT (N = 60)	BCP (N = 400)	ACBP (N = 400)	PCT (N = 206)	PPCT (N = 410)	BCP (N = 179)	PCT (N = 182)
Age, median (range)	62 (41 – 71)	60 (35 – 72)	63 (31 – 90)	63 (31 – 89)	63.5	65.0	65.4 (41.2 – 86.2)	65.8 (38.4 – 84.1)
Sex, female percent	22	30	40.2	40.0	47	38	47	38
Ethnicity, percent								
White	-	-	84	80	-	-	87.7	90.7
Asian	-	-	12	14	-	-	0.0	2.2
Black	-	-	3	1	-	-	11.2	6.0
Other	-	-	2	5	-	-	1.1	1.1
ISS stage 4, percent	95	95	-	-	100	100	100	99.5
ECOG, percent								
0	79	78	45	40	38.8	45.4	46.9	46.7
1	21	22	55	60	60.7	53.9	53.1	52.7
2	0	0	0	0	0.5	0.7	0.0	0.6
PD-L1 $\geq$ 50, percent	-	-	18.3	18.8	34.0	32.2	-	-
Smoking, current or former percent	68	70	81	79	87.9	88.3	96.1	90.1

TABLE C.2 LOOIC model comparison statistics for M-spline models with and without the proportional hazards assumption, computed within each study in the network and overall. Also shown for the second-order fractional polynomial model with repeated −0.5 powers. Lower values of LOOIC indicate better fit. p_{LOO} is an estimate of the effective number of model parameters.

		Non-proportional hazards			
Study	Proportional hazards 7 independent knots	Treatment effects 8 common knots	Treatment effects 11 common knots	Stratified 7 independent knots	Fractional polynomial $(-0.5, -0.5)$
ERACLE	700.5	702.0	702.5	697.3	729.2
IMpower150	2744.8	2740.6	2741.8	2747.6	3418.4
KEYNOTE189	2425.6	2418.9	2418.9	2425.1	2443.3
PRONOUNCE	1622.3	1609.2	1609.3	1619.7	2225.1
**Total**	**7493.1**	**7470.8**	**7472.5**	**7489.8**	**8816.0**
p_{LOO}	21.9	27.5	30.7	32.0	16073.9

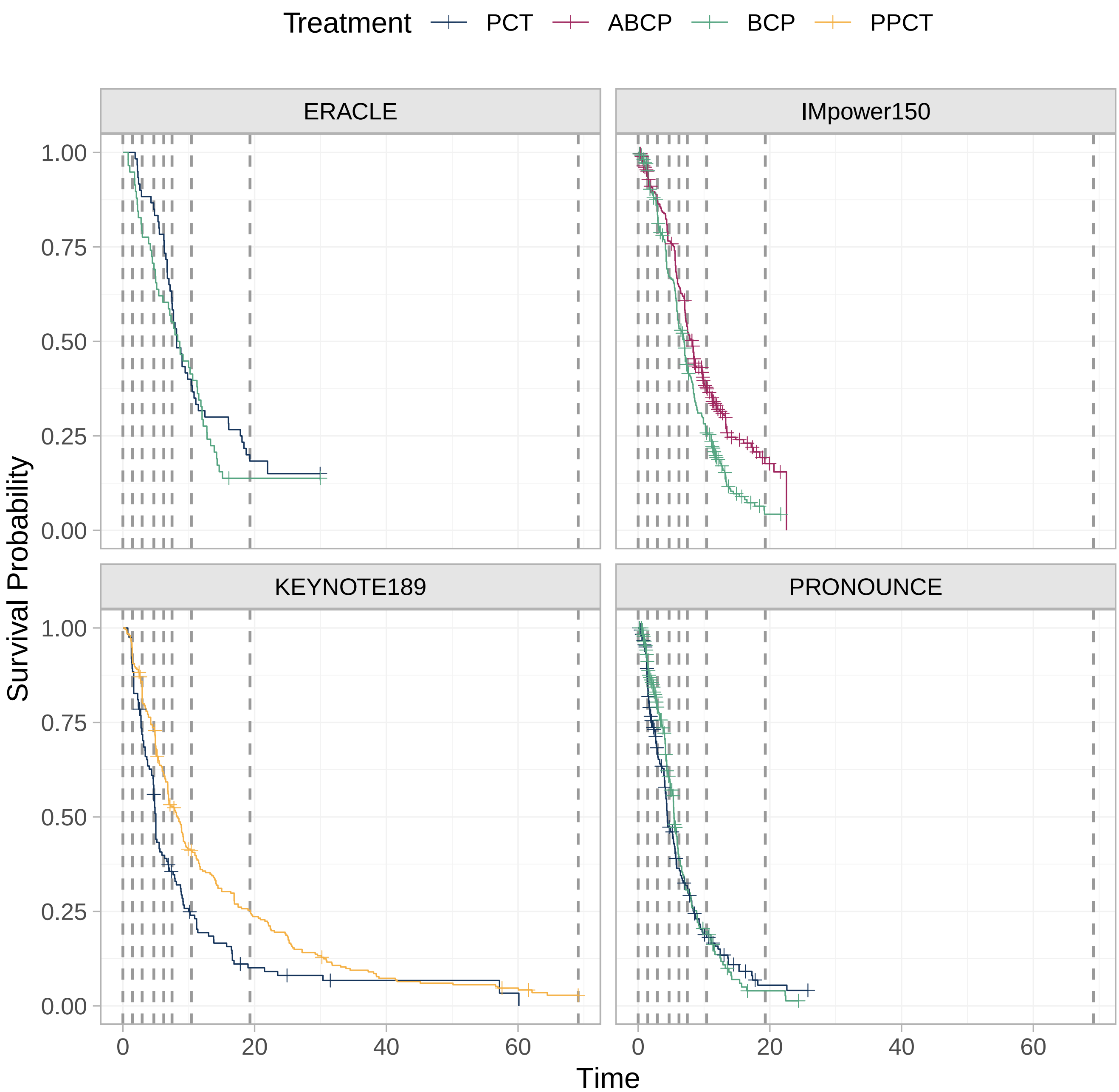

FIGURE C.1 Default common knot locations overlaid on Kaplan-Meier curves in each study population. Seven internal knots are chosen according to quantiles of the quantiles of the observed event times in each study. Follow-up in the IMpower150 and PRONOUNCE studies ends just after the last internal knot, which is likely to lead to sampling issues.

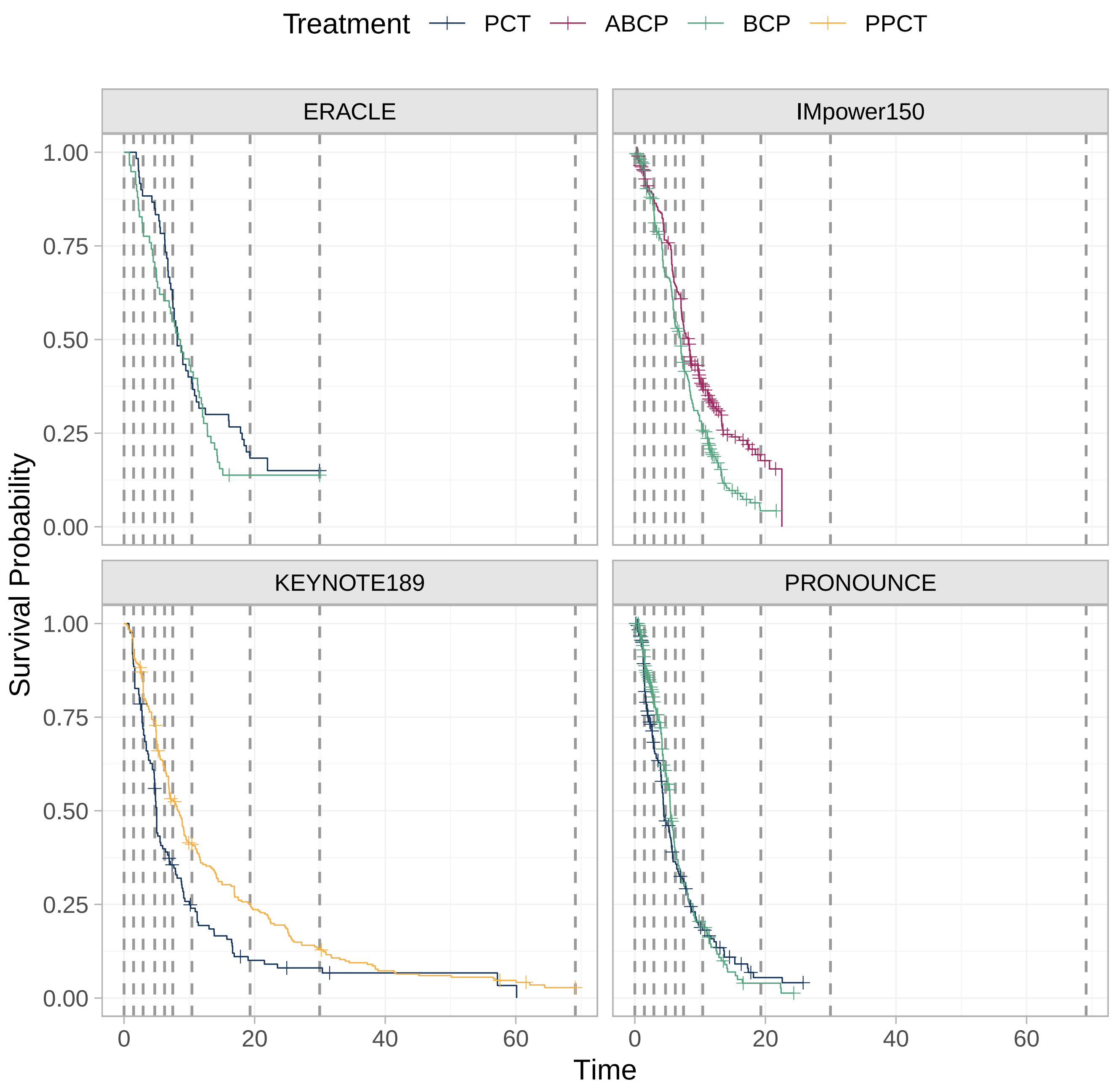

FIGURE C.2 Modified knot locations, with an additional knot added at the end of follow-up of the ERACLE study.

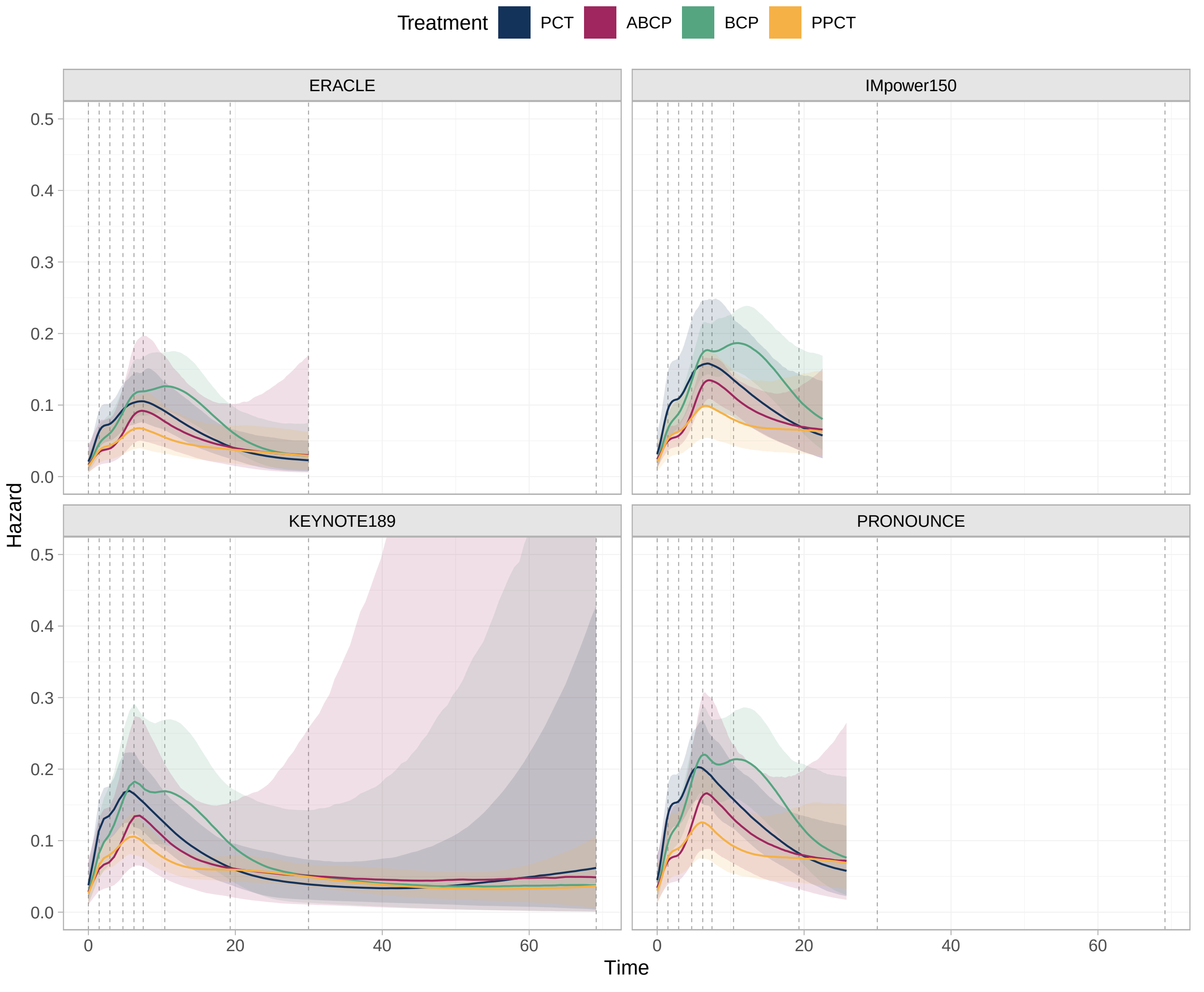

FIGURE C.3 Estimated baseline hazards on each treatment in each study population, from a cubic M-spline model with non-proportional hazards via treatment effects on the spline coefficients. Shaded bands indicate 95% credible intervals. Dashed vertical lines indicate the location of knots.

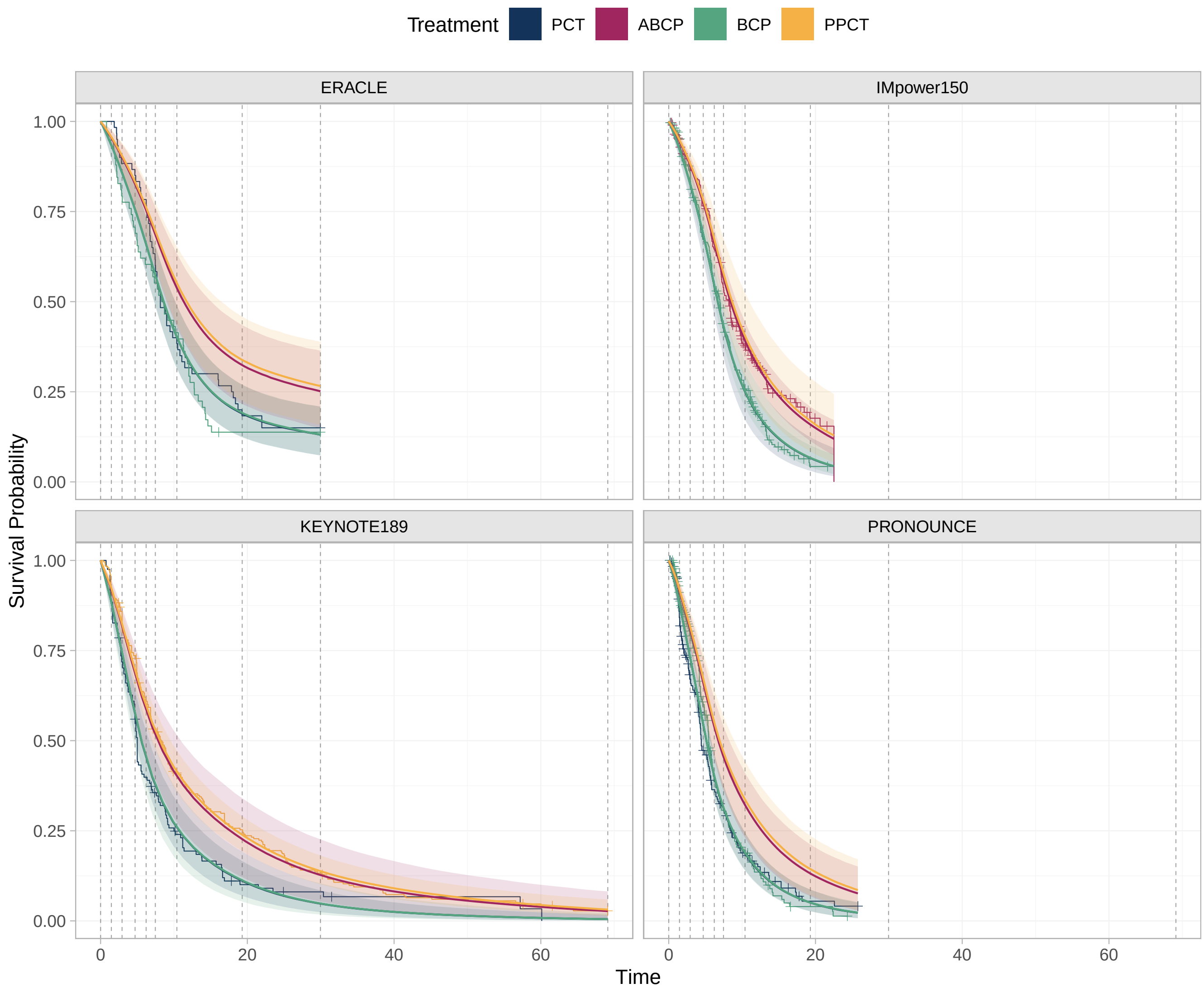

FIGURE C.4 Estimated progression-free survival curves on each treatment in each study population, from a cubic M-spline model with proportional hazards. Shaded bands indicate 95% credible intervals. Note that the estimated PCT and BCP curves are entirely overlapping in this model. Observed Kaplan-Meier curves are also shown. Dashed vertical lines indicate the location of knots.

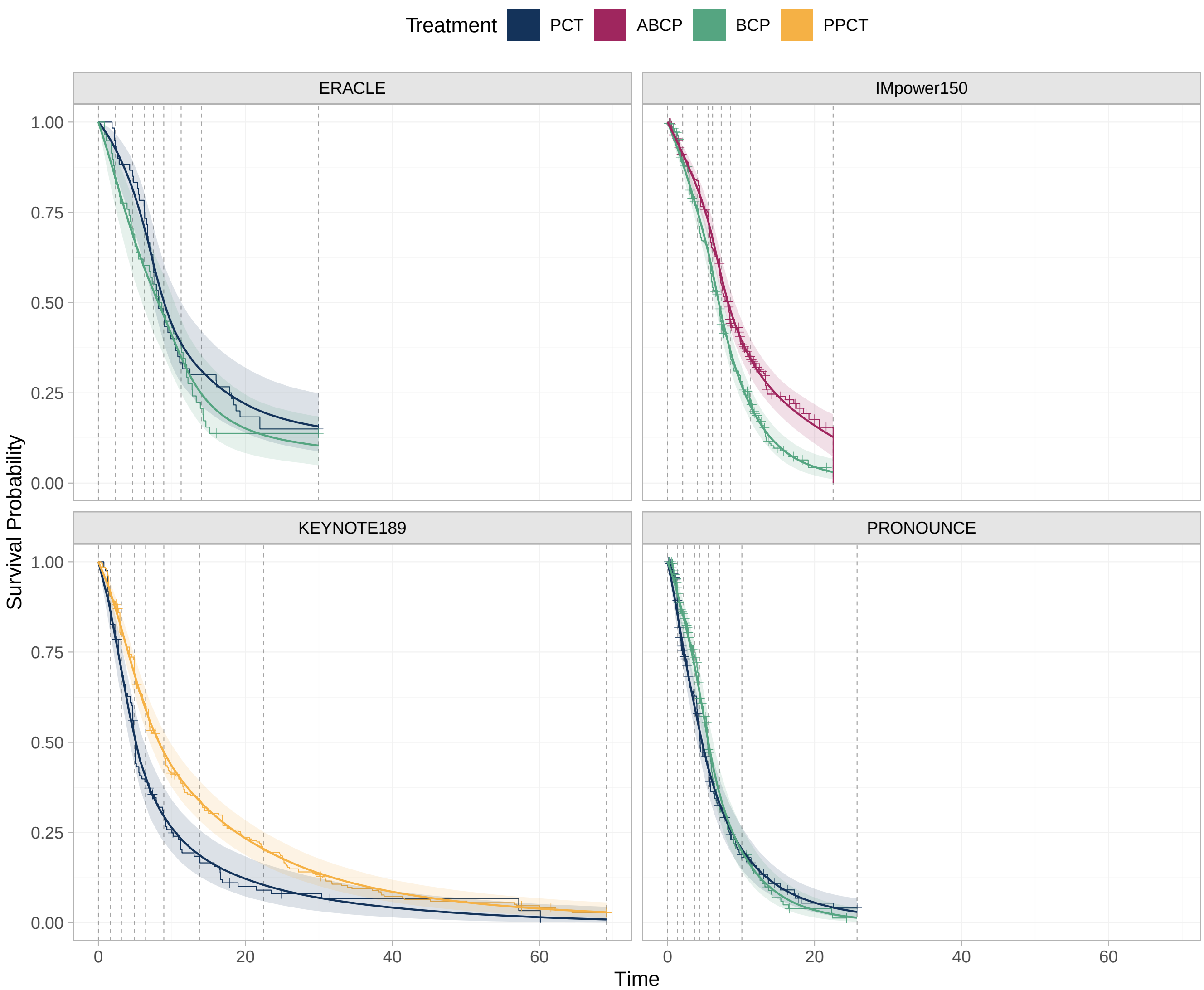

FIGURE C.5 Estimated progression-free survival curves on each treatment in each study population, from a cubic M-spline model with non-proportional hazards via stratified baseline hazards for each treatment arm. Shaded bands indicate 95% credible intervals. Observed Kaplan-Meier curves are also shown. Dashed vertical lines indicate the location of knots.

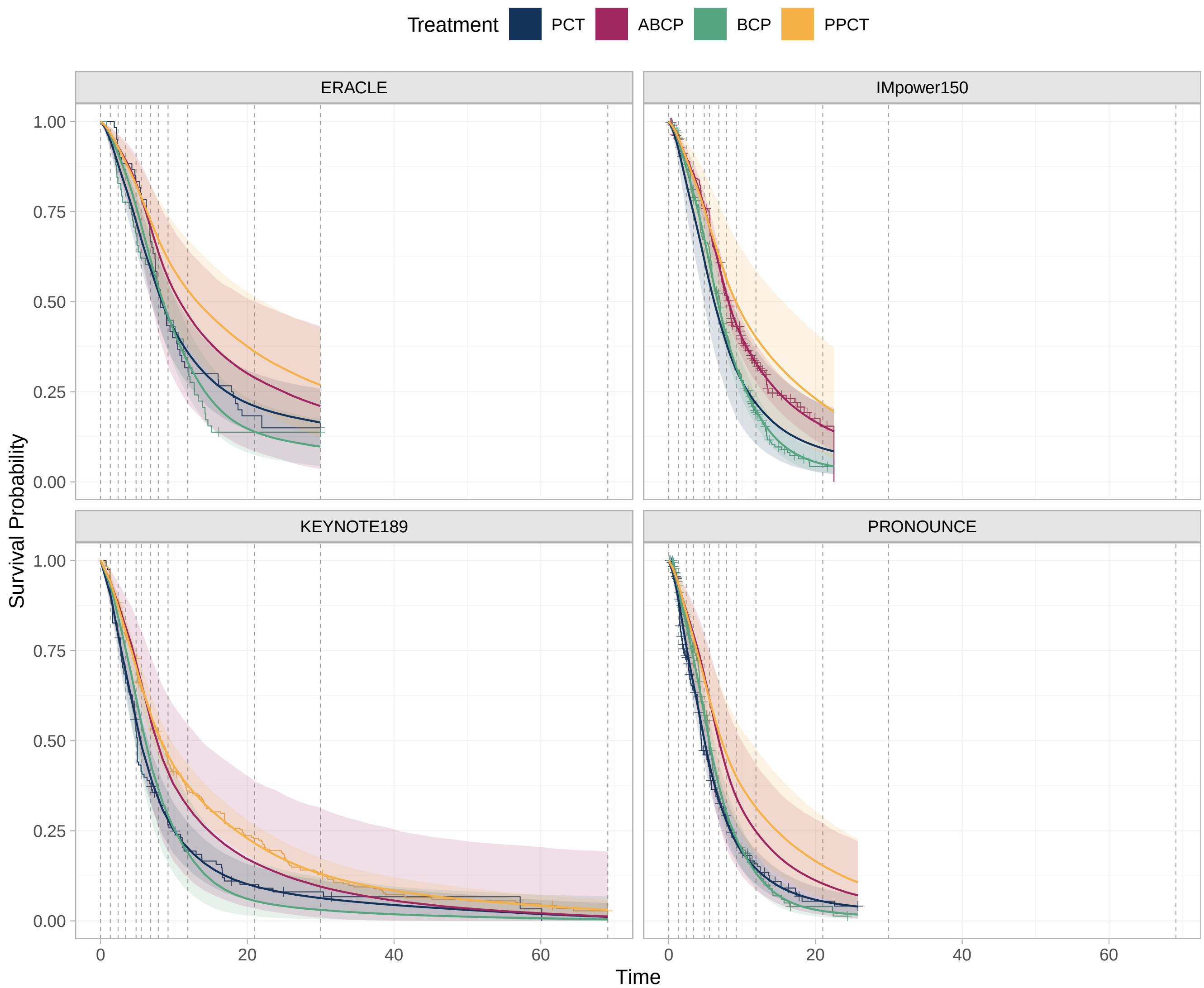

FIGURE C.6 Estimated progression-free survival curves on each treatment in each study population, from a cubic M-spline model with an increased number of knots (11 internal knots). Non-proportional hazards are modelled via treatment effects on the spline coefficients. Shaded bands indicate 95% credible intervals. Observed Kaplan-Meier curves are also shown. Dashed vertical lines indicate the location of knots.

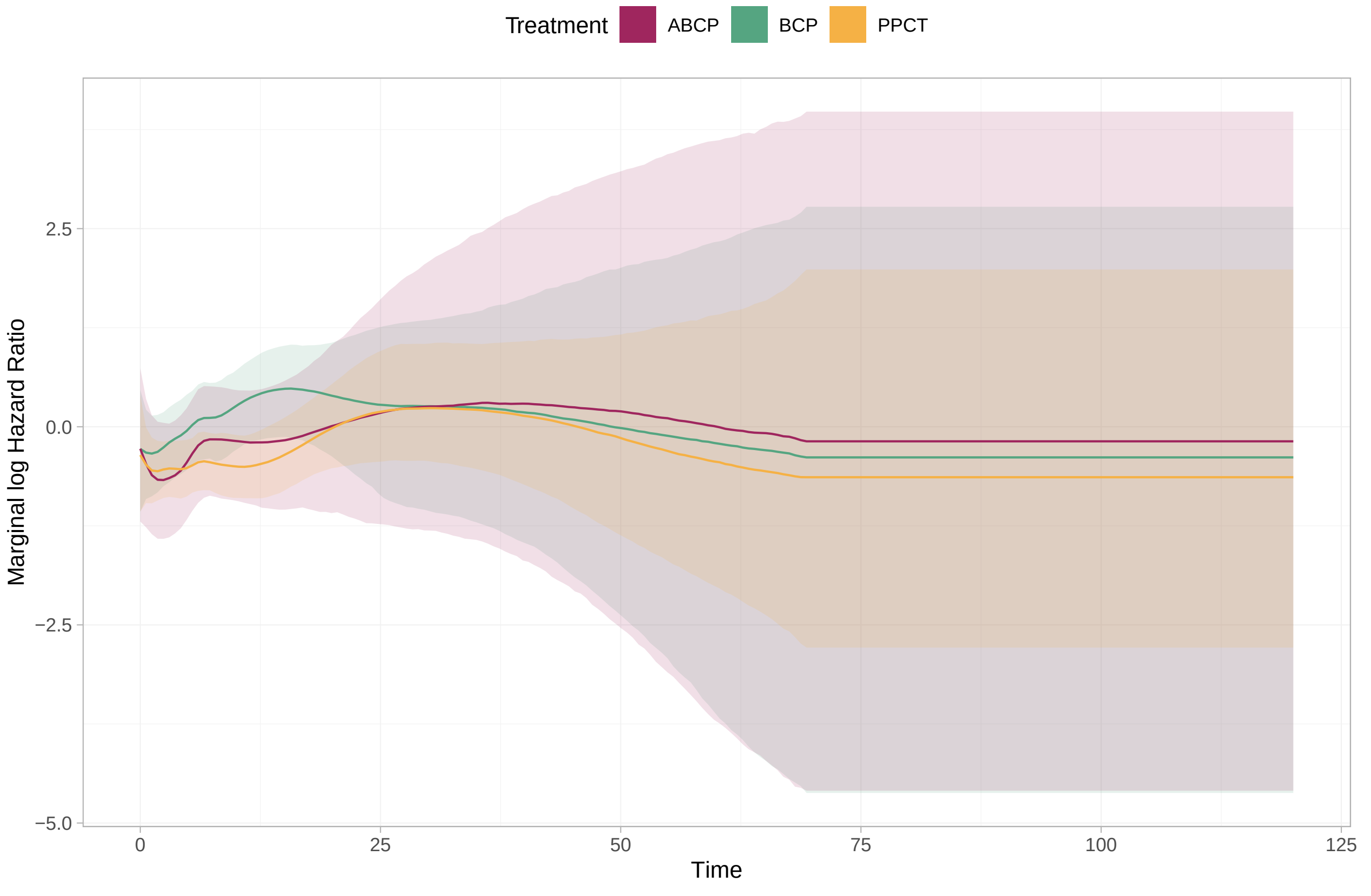

FIGURE C.7 Estimated marginal log hazard ratios on each treatment over time in the KEYNOTE189 population, from a cubic M-spline model with non-proportional hazards via treatment effects on the spline coefficients. Shaded bands indicate 95% credible intervals.

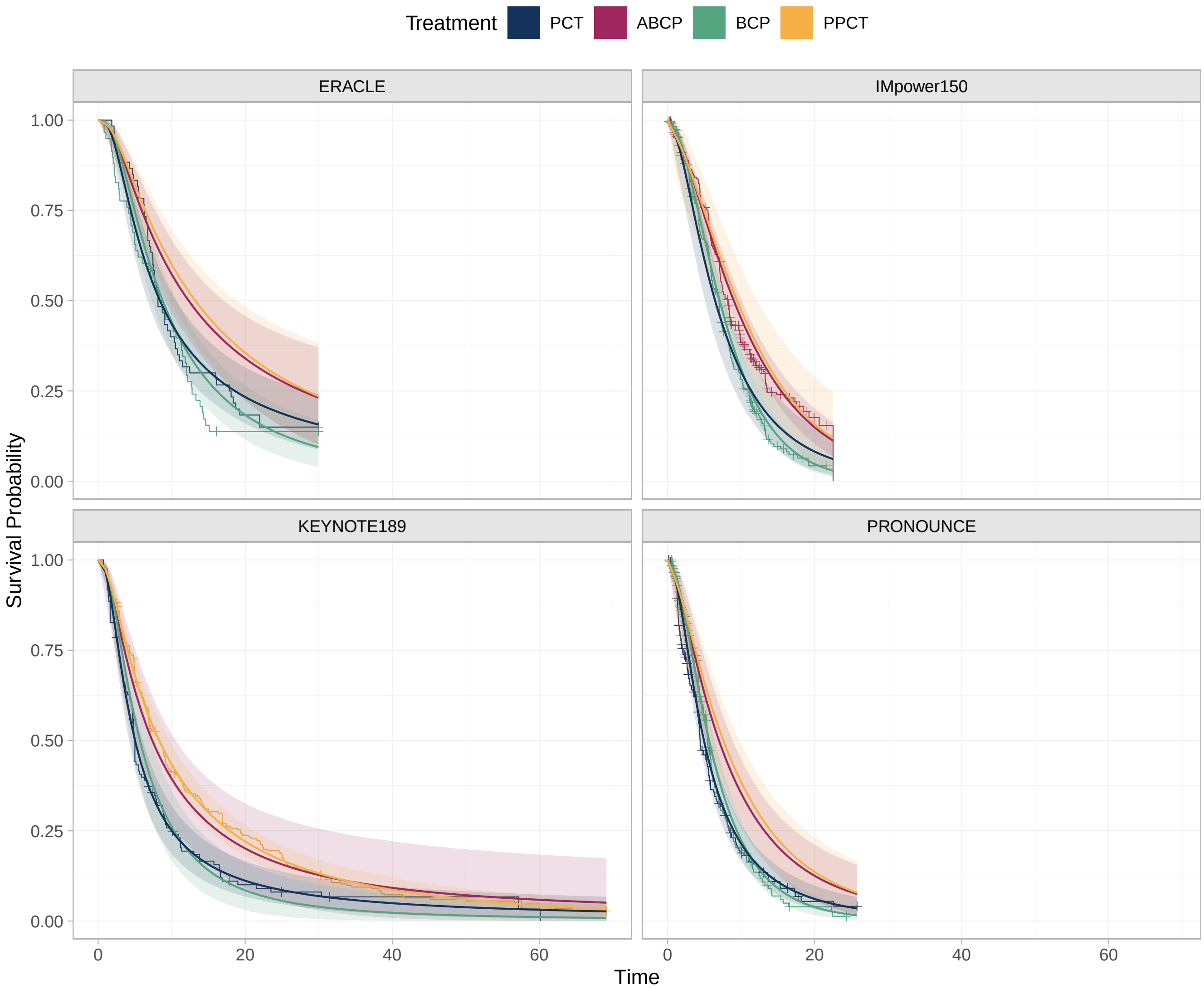

FIGURE C.8 Estimated progression-free survival curves on each treatment, in each study population, from a second-order fractional polynomial model with repeated −0.5 powers. Shaded bands indicate 95% credible intervals. Observed Kaplan-Meier curves are also shown.

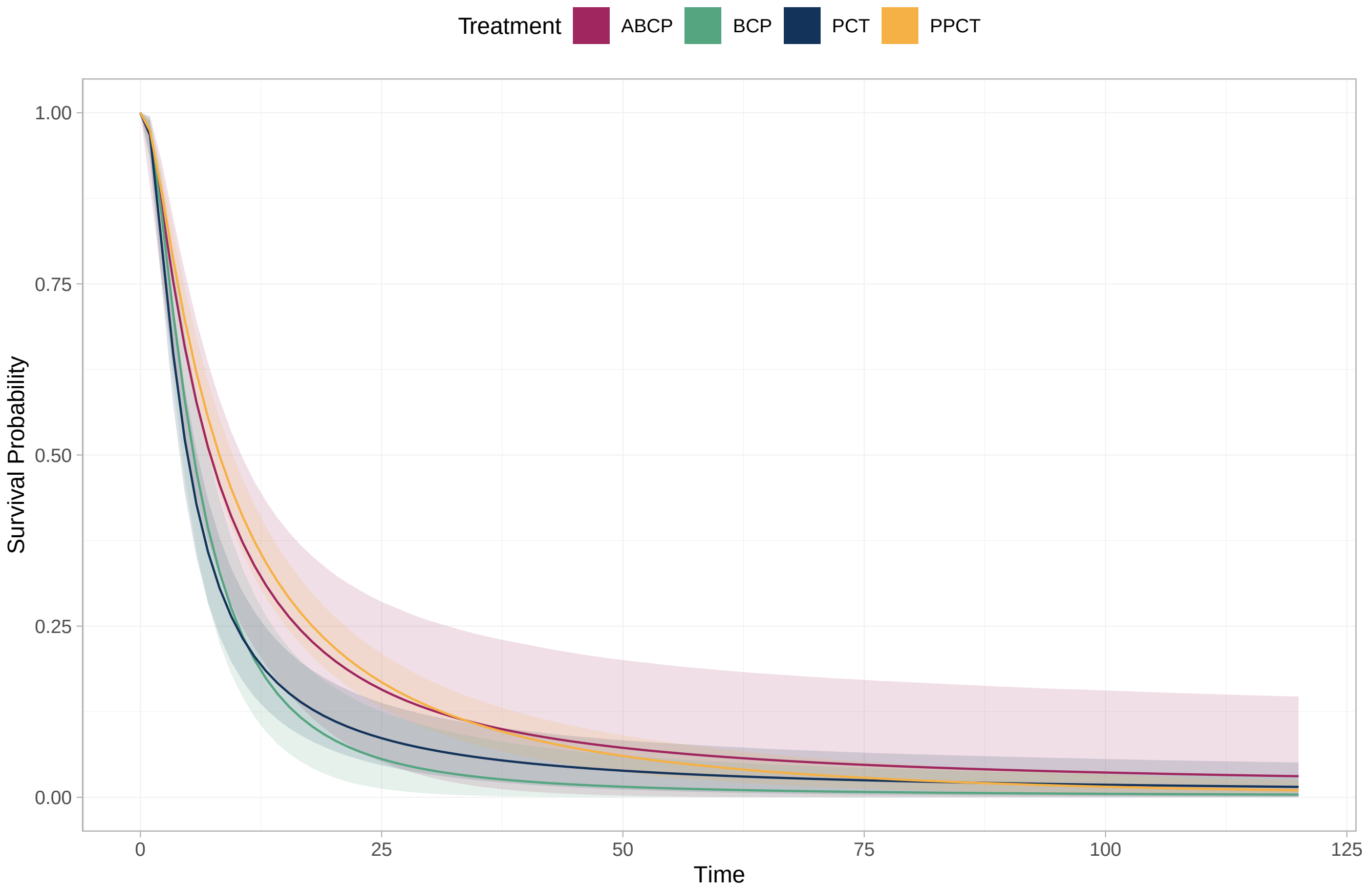

FIGURE C.9 Estimated progression-free survival curves on each treatment in the KEYNOTE189 population, from a second-order fractional polynomial model with repeated -0.5 powers. Shaded bands indicate 95% credible intervals.
